# Development of nursing quality evaluation indicators for extracorporeal membrane oxygenation based on Donabedian theory: A Delphi study

**DOI:** 10.1371/journal.pone.0333410

**Published:** 2025-10-17

**Authors:** Xiaomei Yu, Huihuang Zou, Shuaihong Tong, Yong Zhang, Zhijun Tian, Shichao Zhu

**Affiliations:** Intensive Care Unit, Henan Provincial People’s Hospital, People’s Hospital of Zhengzhou University, Zhengzhou, China; Azienda Ospedaliero Universitaria Careggi, ITALY

## Abstract

Extracorporeal membrane oxygenation (ECMO) serves as a vital therapy for refractory severe respiratory and circulatory failure. With its expanding application in China and the heightened demand for high-quality medical services, ensuring top-notch nursing care during ECMO treatment is crucial. However, existing ECMO nursing quality evaluation tools have limitations, such as lacking detailed calculation formulas for some indicators and insufficient scientific grounding due to over-reliance on expert experience. Thus, there is an urgent need to develop a scientific and practical index system for evaluating ECMO nursing quality to provide an objective basis for monitoring and improving care.This Delphi study was conducted to develop and refine quality indicators for ECMO care, adhering to ACCORD guideline. First, a literature review and expert discussions were carried out to design an expert questionnaire. Then, nineteen experts in critical care participated in two rounds of Delphi surveys. After the surveys, the analytic hierarchy process was employed to assign weights to the indicators.This study achieved a 100 questionnaire recovery rate, and there was high agreement among the experts. Through the two-round consultation, the final ECMO care quality indicators were determined, including three primary indicators, nine secondary indicators, and 32 tertiary indicators. A scientific and reliable ECMO care quality evaluation system was successfully created. This system offers a comprehensive basis for future assessments of ECMO care in China, addressing the need for robust quality evaluation tools in this field and contributing significantly to the continuous improvement of clinical nursing.

## Introduction

ECMO is important for treating refractory severe respiratory and circulatory failure [1]. As ECMO technology matures and social demands for quality healthcare increase, its application is rapidly expanding in China [2]. ECMO is primarily used in critically ill patients as an artificial circulatory and respiratory support device, with risk related to anticoagulation therapy, blood component destruction, systemic inflammatory response, tissue perfusion, and associated invasive procedures [3]. Owing to patients’ hemodynamic instability and underlying diseases, complications, such as pressure injury, hemorrhage, and embolism, are prevalent [4]. Critically ill patients are susceptible to malabsorption, ventilator-associated pneumonia, and catheter-associated tract infections [5]. These complications can worsen patient conditions, prolong illness, and increase resource consumption. ECMO technology usage in clinics is increasing annually but with high technical and operational risks [6]. Effective management strategies, resource allocation, and competent medical staff are essential for successful ECMO implementation, necessitating the comprehensive involvement of nursing staff throughout the ECMO diagnosis and treatment process. These include condition monitoring, patient transfer, surgical assistance, basic care, early rehabilitation, and discharge follow-up, crucial for enhancing patient quality of life and prognosis [7,8]. High-quality nursing care minimizes ECMO-related complications, enhancing the quality of life of patients on ECMO [9]. Developing nursing quality evaluation indicators for ECMO is essential to meet clinical needs and enhance healthcare quality.

Nursing quality is integral to the overall quality of hospital services. Nursing quality management involves assessing and controlling the nursing process to ensure high-quality care [10]. Developing and effectively applying nursing quality evaluation indicators is vital for improving nursing quality management and service quality. These indicators provide quantitative tools to measure nursing quality, support relevant activities [11], and ensure high-quality care across healthcare organizations. The National Nursing Development Plan (2016–2020) emphasizes the importance of establishing mechanisms for quality control and continuous improvement in nursing care. This includes identifying key issues in the nursing process and implementing a system for monitoring and providing regular feedback on indicators to improve nursing quality. Nursing quality evaluation indicators are important checkpoints in the care process, quantifying criteria that affect patient outcomes, and best practices are achieved through the monitoring and intervention of the indicators [12]. By monitoring and intervening on these indicators, best practices can be achieved.

## Background

In the United States, Donabedian [13] developed the theoretical model of a “three-dimensional quality structure.” Donabedian’s three-dimensional quality structure model (structure, process, outcome) provides a framework for developing nursing quality indicators [13]. The American Society of Critical Care Medicine and ELSO recommends forming multidisciplinary teams to review cases, develop quality indicators, and improve ECMO care [14,15]. Following the Delphi consultation with ECMO experts from five continents, the International ECMO Network identified eight core outcome measures: adverse events, death, intracranial hemorrhage, major bleeding, health-related quality of life, neurological recovery, disability, activities of daily living, and return to work [16]. In 2021, the Israeli Ministry of Health formed the Professional Nursing Practice Advisory Committee and gathered 15 ECMO experts to determine care-related quality indicators, including skin integrity, pain assessment, sedation assessment, bowel motility, and temperature management, for patients receiving ECMO. The recommended quality indicators are derived from the clinical expertise of the committee members [17]. Gai et al. [18] constructed a set of 19 sensitive quality indicators for specialized ECMO care through two rounds of written consultations. However, no detailed calculation formula, and indicators, such as ECMO equipment operation monitoring, complication assessment, and weaning assessment, exists. Additionally, indicator construction is mostly based on expert experience and clinical summaries, which lack theoretical guidance and scientific characteristics.

## Methods

This study aimed to identify the quality indicators for ECMO care using the three-dimensional quality structure model and Delphi expert consultations. The goal is to enhance ECMO clinical care and establish a foundation for future research on ECMO care safety and quality management. This study adhered to the ACCORD(ACcurate COnsensus Reporting Document) guideline [19]. A combination of qualitative and quantitative research methods was used to identify ECMO quality of care (QoC) evaluation indicators and obtain weighting factors for each indicator.

## Establishment of research group

We established a team of nine medical staff members, including four associate chief nurses, five supervisor nurses, four with a master’s degree or higher, and five with a bachelor’s degree. The team’s responsibilities included: (i) screening the ECMO nursing training index system; (ii) compiling an expert consultation form; (iii) selecting and identifying corresponding experts; and (iv) collating and analyzing consultation opinions and data.

## Literature source and retrieval method

The research group conducted an extensive search for relevant domestic and international studies, reviewing the references of selected articles. Two trained researchers conducted a comprehensive search of PubMed, MEDLINE, Cochrane Library, OVID, CNKI, and WANGFANG databases for studies published up to September 2022. Keywords included “extracorporeal membrane oxygenation,” “extracorporeal circulation,” “quality indicators,” and “nursing quality.” Relevant articles were selected, and Donabedian’s theory was applied to identify structural, process, and outcome indicators.

## Consultation process

The team reached a consensus on the names, meanings, calculation formulas, and data collection methods for the preliminary indicators. A draft questionnaire was developed and refined through feedback from four additional experts. Two rounds of questionnaires were sent to panel members from May 2023, each lasting 2 weeks, followed by a 1-week break. Final actual completion in July 2023. Each questionnaire, which took approximately 15-25 min to complete, was mailed and returned. The questionnaire included: (i) an introduction outlining the study’s aim, structure, completion requirements, and submission deadline; (ii) definitions and calculation formulas for the candidate indicators (the experts evaluated each indicator’s significance and rationality using a five-point Likert scale [5-very important, 4-important, 3-moderately important, 2-not very important, 1-not important], and a comment column was provided for experts to rephrase or delete indicators, suggest new indicators, and provide qualitative comments; (iii) general information questionnaire on experts (age, educational background, working years, and professional distribution); and (iv) experts’ familiarity with indicators and basis of judgement (familiarity was divided into five levels: very familiar, familiar, average, unfamiliar, and very unfamiliar, with values of 1.0, 0.8, 0.6, 0.4, and 0.2, respectively). The evaluation was based on theoretical analysis, practical experience, domestic and international references, and subjective judgment. All questionnaires were completed individually and returned directly to the research team. Responses were anonymized and aggregated before analysis, ensuring that experts were unaware of others’ scores or comments, thereby minimizing peer pressure and conformity bias. Following the initial questionnaire collection, we adjusted the indicators based on expert opinions, deleting indicators with mean significance<3.50 points or coefficient of variation>0.25. Further, we revised the indicators based on the research, discussion, and expert opinions while revising certain indicator titles and descriptions to convey their importance more accurately. A second round of questionnaires was then prepared. Following the two consultation rounds, expert opinions converged, leading to the consultation’s conclusion.

## Two rounds of expert consultations

Experts from 19 national tertiary hospitals in 11 provinces and cities (five within and six outside the province) in China participated in the consultations. The experts all possessed academic authority, extensive theoretical knowledge, and clinical practice experience in relevant research fields. Strict inclusion criteria were established for the Delphi method experts, including: (i)>10 years of professional experience; (ii) head of the nursing department or intensive care unit (ICU) with involvement in ECMO care or management; (iii) a bachelor’s degree or higher in nursing; (iv) a professional title of supervisor nurse or above; and (v) willingness to participate in multiple rounds of expert consultation. All five inclusion criteria were mandatory for expert participation, ensuring that each panelist possessed extensive professional experience, relevant leadership roles, academic qualifications, and willingness to engage in multiple Delphi rounds.

## Analytic hierarchy process

The analytic hierarchy process (AHP) was employed to determine the relative weights of each indicator [20]. In AHP, each expert conducted pairwise comparisons between indicators within the same hierarchy level using the Saaty 1–9 scale. The mean judgment matrix was calculated across experts, and eigenvector normalization produced the relative weights. The consistency ratio (CR) was calculated, and a CR<0.1 was considered acceptable to ensure logical consistency.

## Statistical analysis

Data analysis, double entry, and verification were conducted using SPSS 24.0 software. Expert profiles were analyzed using frequencies and percentages. Descriptive statistics were reported as mean, standard deviation (SD), and coefficient of variation (CV). Expert authority was calculated as a coefficient of authority (Cr), calculated from their judgement basis (Ca) and familiarity with the consultation content (Cs). The coordination of experts’ opinions was indicated by the CV and Kendall coefficient of coordination (W), reflecting the degree of fluctuation of the opinions. The higher the W value, the higher the agreement between the expert panel members.

## Ethical considerations

This study was approved by the Ethics Committee of Henan Provincial People’s Hospital (No.2023-37). The Ethics Committee waived the requirement for participants to obtain consent. All participants were anonymized and informed about the study’s background, purpose, questionnaire requirements, expected results, and their right to opt-out.

## Results

### Literature search

A total of 1,193 articles were identified during the search process. After removing duplicates (284) and excluding irrelevant articles (909), 30 articles were presented to the review panel.

### Basic information of the experts

Nineteen experts from medical colleges and tertiary hospitals across China (Henan, Beijing, Shanghai, Hunan, and other regions) participated in two interview rounds. Most panel members were female (78.95). The mean age was (45.16±5.81) years, and the mean experience in ECMO was (23.05±6.30) years. Clinical nursing was the most common specialty (68.42). The experts’ titles were distributed as follows: intermediate (15.79), assistant senior (52.63), and senior (31.58) levels. Of the experts, 63.15 held a bachelor’s degree, and 36.85 had a master’s degree or higher (Table 1).

**Table 1:** Two rounds of expert demographic characteristics.

Characteristics		Two rounds	
Sex	Male	7	21.05
	Female	12	78.95
Age range (years)	35–39	2	10.53
	40–44	9	47.37
	45–49	4	21.05
	50–55	4	21.05
Years of working	<20	6	31.58
	20–25	7	36.84
	26–30	3	15.79
	>30	3	15.79
Job title	intermediate level	3	15.79
	deputy senior level	10	52.63
	senior level	6	31.58
Education level	Bachelor’s degree	12	63.15
	Master’s degree	5	26.32
	Doctorater’s degree	2	10.53
Professional field	Nursing management	4	21.05
	ICU	8	42.10
	RICU	2	10.53
	CCU	3	15.79
	Nursing education	2	10.53

### Enthusiasm and authority of the experts

All 19 questionnaires were completed in two rounds, achieving a 100 response rate. In the first round, 15 experts provided constructive feedback, whereas four experts did so in the second round. The experts had a high average authority coefficient of 0.874, an indicator familiarity of 0.963, and an overall authority degree of 0.9185, demonstrating reliability in their consultation (S1 File).

### Consensus on ECMO nursing quality indicators

The Kendall harmony coefficients ranged from 0.111–0.145 (P<0.05) (Table 2).Although the overall Kendall’s W values, were low, the second round’s W value was higher than the first, indicating increasing consensus among the experts.

**Table 2:** Expert opinion coordination levels.

	Importance			Rationality of calculation formula			Feasibility of data collection		
	Kendall W	ChiSquared Value	P Value	Kendall W	ChiSquared Value	P Value	Kendall W	ChiSquared Value	P Value
First round	0.125	83.889	0.000	0.127	63.120	0.001	0.111	65.460	0.000
Second round	0.145	118.72	0.000	0.132	77.590	0.000	0.119	69.939	0.000

### Expert consultation results

In the first round, 15 experts proposed 28 modifications. This study included indicators based on the principle that ECMO is team-managed, emphasizing the QoC and the specialization of the indicators.

The secondary index “nursing adverse events” was added, while the tertiary indexes “incidence of in-hospital stress injury in patients on ECMO” and “unplanned extubation” were adjusted under this category. The experts believed that the three-level index of “ICU nurse ratio” did not reflect the role of nurses in the team, and they suggested changing it to “nurse ratio in the ECMO team.” According to technical specifications issued by the government (2022)[21], the main indicator reflecting ECMO’s therapeutic effect is the “ECMO effective weaning rate.” They also recommended renaming it to “ECMO patient survival rate,” removing “probability of successful weaning from ECMO,” and including “incidence of decreased oxygenator function.” Finally, the indicator calculation methods were revised per experts’ opinions. In the second round, the five experts proposed revisions. The “incidence of limb ischemia on the puncture side” in the patient outcome was renamed “incidence of limb ischemic necrosis,” and the experts felt that the outcome assessment should be more comprehensive. The three-level index “complication risk factor evaluation rate” was adjusted to align with clinical practice. Appendix Tables 3 and 4 present the results of the two survey rounds. The final indicators included three primary, nine secondary, and 32 tertiary indicators.

### Relative importance of indicators

Appendix Table 5 presents the relative weights of the indicators. The process indicator had the highest weight (0.4126) among the primary indicators. Among the secondary indicators, nursing assessment (0.2178) and interventions (0.1372) ranked first and second, respectively. Among the tertiary indicators, ECMO equipment readiness (0.0841), the incidence of decreased oxygenator function (0.0693), and ECMO member credentials (0.0655) were the top three.

## Discussion

This study utilized two rounds of expert correspondence and AHP to develop ECMO nursing quality evaluation indicators that experts deemed feasible and appropriate. The Cr values from both rounds indicated that the 19 experts possessed substantial theoretical knowledge, practical experience, and authority in the field of competence. The experts came from the top three hospitals in 11 provinces and cities across China, ensuring broad representation and nationwide applicability. Both the Kendall W value and harmony coefficient indicate good coordination of expert opinions. Although Kendall’s W values were relatively low, this may reflect heterogeneity in expert backgrounds, institutional protocols, and regional resource availability. Nevertheless, all W values were statistically significant, indicating meaningful convergence of opinions. The experts’ positive coefficient was 100 in both rounds, with experts actively engaged via phone or email, demonstrating their high attention and commitment. The Cr values of the indicators showed good consistency. The Delphi method and AHP are combined to determine the weight of the index, which makes the result more objective and reliable.

Given ECMO’s complexity, high risk, and cost, it should be performed in experienced centers with adequate volume and expertise to ensure safe usage. This study used the three-dimensional quality model [13] as its theoretical framework, emphasizing quality control of the entire care process. Process indicators had the highest weight, underscoring their significance in ensuring clinical effectiveness, while structure and outcome indicators were important.

The structural indicators included three secondary indicators: human resource allocation, ECMO member qualifications and continuing education, and ECMO-related rules and regulations management, which addressed the prerequisites for ECMO implementation. ECMO care is centered on ICU nurses, who provide a range of professional nursing services, such as line pre-fill, bedside monitoring, treatment, and support [7,8,22]. Prior research [7] has examined the implementation of a 1:1 or 1:2 nurse-to-patient ratio in ECMO centers, where trained nurses deliver 24-hour bedside care and line management, consistent with international consensus [3]. To ensure QoC, the experts required a 1:1 nurse-to-patient ratio, consistent with our clinical practice. Studies on Chinese nurses’ knowledge of ECMO care revealed a low-to-medium knowledge base [23], emphasizing the importance of specialized ECMO training [23,24]. Establishing a multidisciplinary team ensures rapid availability of personnel for resuscitation and minimizes patient waiting times [25,26]. The establishment and implementation of regulations ensure the quality of staff training and assessment, crucial for improving the level of ECMO emergency specialization, as demonstrated by the Houston Vascular Centre in the USA [8]. Research suggests that implementing an emergency plan enhances nurses’ crisis awareness [14].

The prioritization of process indicators in this study aligns with Gai et al.’s [18] findings. Emphasizing advanced monitoring of nursing quality allows nurses to provide more anticipatory care, ultimately improving overall nursing quality. The experts believed process evaluation to be key to high-quality ECMO care, reflecting the nursing staff’s operational processes as the primary factor influencing quality. This aligns with some scholars’ views that process indicators are more readily controlled and improved compared with outcome indicators, hence their potentially greater importance [27]. As ECMO’s clinical application involves different modalities, differences exist in its monitoring focus [28]. In this study, various ECMO aspects were refined in the construction of ECMO process indicators, which included three secondary and 13 tertiary indicators. Pre-operative equipment checks ensure proper standby status. Pipeline maintenance includes verifying proper installation, tightness, and looseness or kinks. A smooth pipeline is crucial for normal machine operation. Additionally, patient assessments of each organ system, including limb perfusion, neurological examination, and risk factor evaluations, are conducted to ensure patient safety. Second, the quality of nursing practices significantly impacts ECMO’s success [22,29]. Nurses must meticulously ensure correct tubing filling rates and the absence of air bubbles. Strict adherence to checklists while monitoring ECMO parameters and indicators ensures the effectiveness of adjuvant therapy [30]. Furthermore, monitoring anticoagulation and coagulation indices during pipeline operation is critical. The anticoagulation effect directly impacts patient safety and pipeline lifespan, while monitoring the patient’s coagulation indices guarantees patient safety [26,28]. To protect the safety of patients and medical personnel during the puncture process, the degree of patient sedation should be assessed before the puncture to determine if it reaches the target value, avoiding agitation and potential risks during the procedure [31]. The execution rate of the withdrawal test contributes to improving the success rate of withdrawal, ensuring clinical therapeutic effect and demonstrating the importance of standardizing the process of discharging the machine [32]. Finally, the quality of the ECMO machine can be monitored by determining whether the target temperature and flow rate have been reached [29].

The final outcome indicators in this study, which included three secondary and 11 tertiary indicators, demonstrate the close relationship between nursing quality and patient clinical outcomes. Schindler et al. [33] showed that critically ill children or infants treated with ECMO had an eight-fold higher incidence of stress injury than other ICU patients, associated with plasma protein destruction, hemodilution, and circulatory instability due to ECMO treatment. The experts in this study concluded that machine-related complications during ECMO operations are also important indicators of the quality of specialized care, distinguishing them from other ICU patients with specificity, especially oxygenators and centrifugal pumps. Complications during ECMO therapy significantly affect patients’ long-term prognosis and quality of life. The outcome indicators of this study were related to the incidence of six common complications associated with ECMO treatment (hemorrhage, ischemic necrosis, hemolysis, thrombosis, and ECMO catheter bloodstream infections), with hemorrhage having the highest incidence. Bleeding is a common complication of ECMO treatment [34], and previous studies in China have shown that bleeding incidence during ECMO therapy ranges from 12.0–52.0 owing to stress ulcers and inappropriate use of anticoagulants [35]. A Korean study found that delirium in patients on ECMO is also a factor that affects their quality of life and can be used as an outcome indicator [36], consistent with the results of a systematic evaluation in Poland [37]. The experts in this study concluded that delirium in patients on ECMO in an ICU is not specifically related to their treatment. Moreover, adding satisfaction evaluations can provide a multidimensional assessment of nursing care quality and help nurses take the initiative to improve themselves [14,18]. Experts concur that clinical complications are crucial for assessing patient prognosis and that nurses can enhance ECMO specialty care quality through targeted interventions.

## Limitations

First, some indicators, such as “ECMO-related emergency plans,” lack uniform quantitative standards across regions and hospitals. Specific improvements should be made to its content regarding specific circumstances. Second, In addition to data feasibility, future studies should systematically evaluate the resource feasibility of each indicator, such as the availability of adequately trained ECMO nursing staff, to ensure practical implementation. Third, this study has not yet been clinically applied, and the sensitivity and validity of its indicators need to be tested. Additionally, as this study was conducted exclusively in China, the generalizability of the indicators to other national healthcare contexts may be limited. Additional validation in diverse international settings is warranted. Furthermore, we predefined two Delphi rounds to balance methodological rigor with feasibility and expert workload. Although all indicators reached consensus by the second round, some items showed substantial changes in agreement between rounds. This may have limited further refinement, and future studies could adopt adaptive rounds based on consensus variability. Lastly, during the correspondence process, both researchers and experts found that ECMO, as a technology carried out in cooperation between healthcare and nursing, has many outcome indicators that correlate with both nursing and medical treatment qualities. These indicators are insufficient to solely evaluate nursing quality. Therefore, it is necessary that the indicators be further improved through clinical practice, adjusted for weak links in the nursing process, and quality control should be carried out throughout the treatment process.

## Clinical relevance

The nursing quality evaluation indicators included quantitative measures of nursing quality, tools for evaluating nursing quality and care activities, and important tools for managing nursing quality. This study’s ECMO quality indicators, developed using the Delphi method, include structural, outcome, and process indicators, with detailed descriptions of the names, calculation formulas, and data collection methods. These indicators offer a comprehensive foundation for clinical practice and allow care managers to adjust data collection methods for more accurate evaluations of ECMO nursing quality.

## Conclusion

This study utilized the Delphi method to develop an ECMO nursing quality indicator system, based on the structure-process-outcome three-dimensional quality model. These indicators are scientific, reliable, and practical, and can be used as the basis for the assessment of ECMO efficacy in patients, and a quantitative evaluation tool for ECMO-specialized nursing services.

## Supporting information

Appendix Tables 3–5. (DOCX)

S1 File Data. (ZIP)
