# Supplementary material for: Development of nursing quality evaluation indicators for extracorporeal membrane oxygenation based on Donabedian theory: A Delphi study
Source: PLoS One. 2025 Oct 17;20(10):e0333410. doi: 10.1371/journal.pone.0333410 (PMC12533855; doi:10.1371/journal.pone.0333410)
Supplement: Appendix — (DOCX) [file pone.0333410.s001.docx]

**Tables**

**Table 3. The proposed indicators and their revisions following two rounds of expert consultation.**

| **Indicator** | **First round** | | | | | **Second round** | | | | |
| --- | --- | --- | --- | --- | --- | --- | --- | --- | --- | --- |
|  | M±SD | CV | Percentage for full score (%) | Outcome | Revised/added  Indicator | M±SD | CV | Percentage for full score (%) | Outcome | Revised/ added  indicator |
| 1. Structure | 4.47±0.77 | 0.17 | 63.2 | Accepted |  | 4.89±0.32 | 0.07 | 89.5 | Accepted |  |
| 1.1 Human resource allocation | 4.89±0.32 | 0.07 | 89.5 | Accepted |  | 4.84±0.50 | 0.10 | 89.5 | Accepted |  |
| 1.1.1 Ratio of ECMO nurse to patient | 4.74±0.45 | 0.09 | 73.7 | Accepted |  | 4.53±0.61 | 0.13 | 57.9 | Accepted |  |
| 1.1.2 Formation of ECMO multidisciplinary team | 4.79±0.42 | 0.09 | 78.9 | Accepted |  | 4.32±0.48 | 0.11 | 68.4 | Accepted |  |
| 1.1.3 Proportion of ICU nurses | 4.53±0.70 | 0.15 | 63.2 | Revised | Percentage of nurses in ECMO teams | 4.63±0.50 | 0.11 | 63.2 | Accepted |  |
| 1.2 ECMO membership qualification and continuing education | 4.79±0.54 | 0.11 | 84.2 | Accepted |  | 4.84±0.37 | 0.08 | 84.2 | Accepted |  |
| 1.2.1 Rate of ECMO members’ credentials | 4.63±0.68 | 0.15 | 73.7 | Accepted |  | 4.53±0.61 | 0.13 | 57.9 | Accepted |  |
| 1.2.2 Rate of training qualified of ECMO members | 4.53±0.77 | 0.17 | 68.4 | Accepted |  | 4.53±0.61 | 0.130 | 57.9 | Accepted |  |
| 1.3 ECMO related rules and regulations management | 4.74±0.56 | 0.12 | 78.9 | Accepted |  | 4.63±0.60 | 0.13 | 68.4 | Accepted |  |
| 1.3.1 Rate of qualified management of ECMO nursing routine | 4.58±0.69 | 0.15 | 68.4 | Accepted |  | 4.58±0.69 | 0.15 | 68.4 | Accepted |  |
| 1.3.2 Rate of qualified management of ECMO nursing practice | 4.74±0.56 | 0.12 | 78.9 | Accepted |  | 4.58±0.61 | 0.13 | 63.2 | Accepted |  |
| 1.3.3 Rate of qualified management of ECMO-related emergency plans | 4.68±0.58 | 0.12 | 73.7 | Accepted |  | 4.84±0.37 | 0.08 | 84.2 | Accepted |  |
| 2. Process | 4.79±0.72 | 0.15 | 78.9 | Accepted |  | 4.89±0.32 | 0.07 | 89.5 | Accepted |  |
| 2.1 Nursing assessment | 4.79±0.54 | 0.11 | 84.2 | Accepted |  | 4.79±0.42 | 0.09 | 78.9 | Accepted |  |
| 2.1.1 Rate of ECMO equipment’s readiness | 4.79±0.54 | 0.11 | 84.2 | Accepted |  | 4.84±0.37 | 0.08 | 84.2 | Accepted |  |
| 2.1.2 Rate of implementation of blood transport assessment of limb | 4.68±0.58 | 0.12 | 73.7 | Accepted |  | 4.58±0.69 | 0.15 | 68.4 | Accepted |  |
| 2.1.3 Rate of skin integrity assessment | 4.21±1.08 | 0.26 | 52.6 | Deleted |  |  |  |  |  |  |
| 2.1.4 Rate of qualified ECMO pipeline maintenance | 4.74±0.56 | 0.12 | 78.9 | Accepted |  | 4.74±0.65 | 0.14 | 84.2 | Accepted |  |
| 2.1.5 Rate of implementation of assessment of neurological changes | 4.68±0.58 | 0.12 | 73.7 | Accepted |  | 4.57±0.77 | 0.17 | 47.4 | Accepted |  |
| 2.1.6 Rate of implementation of complication risk factor assessment | 4.37±0.83 | 0.19 | 57.9 | Accepted |  | 4.42±0.61 | 0.14 | 47.4 | Accepted |  |
| 2.2 Nursing measures | 4.89±0.32 | 0.07 | 89.5 | Accepted |  | 4.89±0.32 | 0.07 | 89.5 | Accepted |  |
| 2.2.1 Rate of implementing ECMO verification checklists | 4.63±0.60 | 0.13 | 68.4 | Accepted |  | 4.58±0.69 | 0.15 | 68.4 | Accepted |  |
| 2.2.2 Rate of target blood flow compliance | 4.42±0.84 | 0.19 | 63.2 | Revised | Included 2.3.2 | 4.89±0.32 | 0.07 | 89.5 | Accepted |  |
| 2.2.3 Rate of implementation of transport safety of patients on ECMO | 4.53±0.70 | 0.15 | 63.2 | Accepted |  | 4.47±0.90 | 0.20 | 68.4 | Accepted |  |
| 2.2.4 Rate of qualified pipe prefilling | 4.42±0.77 | 0.17 | 57.9 | Accepted |  | 4.68±0.48 | 0.10 | 68.4 | Accepted |  |
| 2.2.5 Rate of anti-coagulation target attainment | 4.37±0.96 | 0.22 | 63.2 | Accepted |  | 4.26±0.45 | 0.11 | 47.4 | Accepted |  |
| 2.2.6 Rate of analgesia and sedation reaching the standard | 4.53±0.84 | 0.19 | 73.7 | Accepted |  | 4.68±0.58 | 0.12 | 73.7 | Accepted |  |
| 2.2.7 Rate of capacity correct monitoring | 4.21±1.13 | 0.27 | 57.9 | Deleted |  |  |  |  |  |  |
| 2.2.8 Rate of execution of withdrawal test | 4.21±0.85 | 0.20 | 47.4 | Accepted |  | 4.26±0.73 | 0.17 | 47.4 | Accepted |  |
| 2.3 ECMO machine operation quality | 4.74±0.56 | 0.19 | 78.9 | Accepted |  | 4.37±0.50 | 0.11 | 63.2 | Accepted |  |
| 2.3.1 Rate of target temperature attainment | 4.63±0.68 | 0.15 | 73.3 | Accepted |  | 4.58±0.69 | 0.15 | 68.4 | Accepted |  |
| 3. Outcome | 4.74±0.56 | 0.12 | 78.9 | Accepted |  | 4.58±0.61 | 0.13 | 63.2 | Accepted |  |
| 3.1 ECMO-related complications | 4.42±0.61 | 0.14 | 47.4 | Accepted |  | 4.53±0.61 | 0.13 | 63.2 | Accepted |  |
| 3.1.1 Incidence of centrifugal pump failure | 4.42±0.90 | 0.20 | 68.2 | Accepted |  | 4.42±0.51 | 0.16 | 57.9 | Accepted |  |
|  |  |  |  | Added | Incidence of decreased oxygenator function (3.1.2) | 4.21±0.42 | 0.10 | 47.4 | Accepted |  |
| 3.1.2 Incidence of unplanned extubation in patients on ECMO | 4.68±0.67 | 0.14 | 78.9 | Revised | Included 3.3.1 | 4.68±0.48 | 0.10 | 68.4 | Accepted |  |
| 3.1.3 Incidence of pressure injury in patients on ECMO | 4.42±0.90 | 0.20 | 63.2 | Revised | Included 3.3.2 | 4.74±0.45 | 0.09 | 73.7 | Accepted |  |
| 3.2 Clinical outcome of patients | 4.79±0.42 | 0.09 | 78.9 | Accepted |  | 4.68±0.58 | 0.12 | 73.7 | Accepted |  |
| 3.2.1 Incidence of bleeding | 4.58±0.69 | 0.15 | 68.4 | Accepted |  | 4.42±0.61 | 0.14 | 47.4 | Accepted |  |
| 3.2.2 Incidence of limb thrombosis on the puncture site | 4.47±0.90 | 0.20 | 68.4 | Accepted |  | 4.68±0.58 | 0.12 | 73.7 | Revised | Incidence of limb ischemia |
| 3.2.3 Incidence of hemolysis | 4.47±0.90 | 0.20 | 68.4 | Accepted |  | 4.53±0.51 | 0.11 | 52.6 | Accepted |  |
| 3.2.4 Incidence of thrombosis | 4.42±0.90 | 0.20 | 63.2 | Accepted |  | 4.47±0.51 | 0.11 | 47.4 | Accepted |  |
| 3.2.5 Incidence of ECMO catheter bloodstream infection | 4.53±0.90 | 0.19 | 73.7 | Accepted |  | 4.89±0.32 | 0.07 | 89.5 | Accepted |  |
| 3.2.6 Rate of ECMO successful withdrawal | 4.53±0.70 | 0.15 | 63.2 | Revised | Rate of ECMO effective removal | 4.74±0.45 | 0.09 | 73.7 | Accepted |  |
| 3.2.7 Rate of survival of patients undertaking ECMO | 4.11±1.24 | 0.30 | 57.9 | Deleted |  |  |  |  |  |  |
| 3.2.8 Patients’ and family members’ average satisfaction with nursing | 4.47±0.96 | 0.21 | 73.7 | Accepted |  | 4.68±0.48 | 0.10 | 68.4 | Accepted |  |
|  |  |  |  | Added | Nursing adverse events (3.3) | 4.68±0.58 | 0.12 | 73.7 | Accepted |  |

**Table 4. Nursing quality evaluation indicators for ECMO.**

| **First-level indicators** | **Second-level indicators** | **Third-level indicators** | | |
| --- | --- | --- | --- | --- |
|  |  | **Indicators** | **Formula for calculation/Evaluation criterion** | **Data collection method** |
| Structure | Human resource allocation | Ratio of ECMO nurse-patient | $\frac{\text{Number of nurses caring ECMO patients on duty}}{\text{Number of ECMO patients during the statistical period}}\times\text{100\%}$ | Obtained from the hospital human resources department |
|  |  | Formation of ECMO multidisciplinary team | $\frac{\text{Hold a multidisciplinary team in each unit}}{\text{Total number of spot checks in medical units performing ECMO treatment }}\times\text{100\%}$ | Obtained from the medical records and on-the-spot checks |
|  |  | Percentage of nurses in ECMO teams | $\frac{\text{Number of nurses in each team}}{\text{Total number of ECMO team in this unit}}\times\text{100\%}$ | Obtained from the hospital human resources department |
|  | ECMO membership qualification and continuing education | Rate of ECMO members’ credentials | $\frac{\text{Number of members hold certain certificates in each team }}{\text{ Total number of members in each team}}\times\text{100\%}$ | Obtained from the hospital human resources department |
|  |  | Rate of training qualified ECMO members | $\frac{\text{ Number of ECMO members passing the training assessment per quarter}}{\text{Total number of receiving the training assessment per quarter }}\times\text{100\%}$ | Obtained from records of ECMO examination record |
|  | ECMO-related rules and regulations management | Rate of qualified management of ECMO nursing routine | $\frac{\text{Number of qualified management of ECMO nursing routine}}{\text{Total times of spot checks during the statistical period}}\times\text{100\%}$ | Obtained from on-the-spot checks |
|  |  | Rate of qualified management of ECMO nursing practice | $\frac{\text{Times of qualified management of ECMO nursing practice }}{\text{Total times of spot checks during the statistical period}}\times\text{100\%}$ | Obtained from on-the-spot checks |
|  |  | Rate of qualified management of ECMO-related emergency plans | $\frac{\text{Times of qualified management of ECMO related emergency plans}}{\text{Total times of spot checks during the statistical period}}\times\text{100\%}$ | Obtained from on-the-spot checks |
| Process | Nursing assessment | Rate of ECMO equipment readiness | $\frac{\text{Times of ECMO equipment readiness}}{\text{Total times of spot checks during the statistical period}}\times\text{100\%}$ | Obtained from on-the-spot checks |
|  |  | Rate of implementation of blood transport assessment of limb | $\frac{\text{ Times of implement of blood transport assessment of limb}}{\text{Total times of spot checks during the statistical period}}\times100\%$ | Obtained from on-the-spot checks |
|  |  | Rate of qualified ECMO pipeline maintenance | $\frac{\text{Times of qualified of ECMO pipeline maintenance}}{\text{Total times of spot checks during the statistical period}}\times\text{100\%}$ | Obtained from on-the-spot checks |
|  |  | Rate of implementation of assessment of neurological changes | $\frac{\text{Times of implement of assessment of neurological changes}}{\text{Total times of spot checks during the statistical period}}\times\text{100\%}$ | Obtained from on-the-spot checks |
|  |  | Rate of implementation of complication risk factor assessment | $\frac{\text{Times of implement of complication risk factor assessment}}{\text{Total times of spot checks during the statistical period}}\times\text{100\%}$ | Obtained from on-the-spot checks |
|  | Nursing measures | Rate of implementing ECMO verification checklists | $\frac{\text{Times of implement ECMO verification checklists}}{\text{Total times of spot checks during the statistical period}}\times100\%$ | Obtained from on-the-spot checks |
|  |  | Rate of implementation of transport safety of ECMO patients | $\frac{\text{Times of implement of transport safety of ECMO patients}}{\text{Total times of spot checks during the statistical period}}\times100\%$ | Obtained from on-the-spot checks |
|  |  | Rate of qualified pipe prefilling | $\frac{\text{Times of qualified of pipe prefilling}}{\text{Total times of spot checks during the statistical period}}\times\text{100\%}$ | Obtained from on-the-spot checks |
|  |  | Rate of anti-coagulation target attainment | $\frac{\text{Times of anti-coagulation target attainment}}{\text{Total times of spot checks during the statistical period}}\times\text{100\%}$ | Obtained from on-the-spot checks |
|  |  | Rate of analgesia and sedation reaching the standard | $\frac{\text{Times of nalgesia and sedation reaching the standard}}{\text{Total times of spot checks during the statistical period}}\times\text{100\%}$ | Obtained from on-the-spot checks |
|  |  | Rate of execution of withdrawal test | $\frac{\text{Times of execution of withdrawal test }}{\text{Total times of spot checks during the statistical period}}\times\text{100\%}$ | Obtained from on-the-spot checks |
|  | ECMO machine operation quality | Rate of target temperature attainment | $\frac{\text{Times of target temperature attainment}}{\text{Total times of spot checks during the statistical period}}\times\text{100\%}$ | Obtained from on-the-spot checks |
|  |  | Rate of target blood flow compliance | $\frac{\text{Times of target blood flow compliance}}{\text{Total times of spot checks during the statistical period}}\times100\%$ | Obtained from on-the-spot checks |
| Outcome | ECMO-related complications | Incidence of centrifugal pump failure | $\frac{\text{Number of new cases of centrifugal pump failure }}{\text{Total number of ECMO patients during the statistical period }}\times\text{100\%}$ | Obtained from the medical records |
|  |  | Incidence of decreased oxygenator function | $\frac{\text{Number of new cases of unplanned extubation}}{\text{Total number of ECMO patients during the statistical period}}\times\text{100\%}$ | Obtained from the medical records |
|  | Clinical outcome of patients | Incidence of bleeding | $\frac{\text{Number of new cases of bleeding}}{\text{Total number of ECMO patients during the statistical period}}\times\text{100\%}$ | Obtained from the medical records |
|  |  | Incidence of limb ischemia | $\frac{\text{Number of new cases of limb ischemia}}{\text{Total number of ECMO patients during the statistical period}}\times\text{100\%}$ | Obtained from the medical records |
|  |  | Incidence of hemolysis | $\frac{\text{Number of new cases of hemolysis}}{\text{Total number of ECMO patients during the statistical period }}\times\text{100\%}$ | Obtained from the medical records |
|  |  | Incidence of thrombosis | $\frac{\text{Number of new cases of thrombosis}}{\text{Total number of ECMO patients during the statistical period }}\times\text{100\%}$ | Obtained from the medical records |
|  |  | Incidence of ECMO catheter bloodstream infection | $\frac{\text{Number of ECMO-related bloodstream infections}}{\text{Total days of ECMO catheter indwelling in patients during thestatistical period}}\times\text{100\%}$ | Obtained from the medical records |
|  |  | Rate of ECMO effective removal | $\frac{\text{Number of effective ECMO removal cases}}{\text{Total number of ECMO applications during the statistical period }}\times\text{100\%}$ | Obtained from the medical records |
|  |  | Patients’ and family members’ average satisfaction with nursing care | $\frac{\text{Average score of patients and family members evaluation of nursing care during the statistical period }}{\text{Full score of questionnaire}}\times\text{100\%}$ | Obtained from the questionnaire |
|  | Nursing adverse events | Incidence of unplanned extubation in patients on ECMO | $\frac{\text{Number of new cases of decreased oxygenator function}}{\text{Total number of ECMO patients during the statistical period}}\times\text{100\%}$ | Obtained from the medical records |
|  |  | Incidence of pressure injury in patients on ECMO | $\frac{\text{Number of new cases of stress injury}}{\text{Total number of ECMO patients during the statistical period}}\times\text{100\%}$ | Obtained from the medical records（nursing records） |

**Table 5. Relative importance of ECMO nursing quality evaluation indicators (rank by weight).**

|  | **Indicator** | **Weight** |
| --- | --- | --- |
| Primary indicators | Structure | 0.3275 |
|  | Process | 0.4126 |
|  | Outcome | 0.2599 |
| Secondary indicators | Human resource allocation | 0.1310 |
|  | ECMO membership qualification and continuing education | 0.1310 |
|  | ECMO-related rules and regulations management | 0.0655 |
|  | Nursing assessment | 0.2178 |
|  | Nursing measures | 0.1372 |
|  | ECMO machine operation quality | 0.0576 |
|  | ECMO-related complications | 0.0520 |
|  | Clinical outcome of patients | 0.1040 |
|  | Nursing adverse events | 0.1040 |
| Tertiary indicators | Ratio of ECMO nurse to patient | 0.0632 |
|  | Formation of ECMO multidisciplinary team | 0.0459 |
|  | Percentage of nurses in ECMO teams | 0.0218 |
|  | Rate of ECMO members' credentials | 0.0655 |
|  | Rate of training qualified ECMO members | 0.0655 |
|  | Rate of qualified management of ECMO nursing routine | 0.0281 |
|  | Rate of qualified management of ECMO nursing practice | 0.0281 |
|  | Rate of qualified management of ECMO-related emergency plans | 0.0094 |
|  | Rate of ECMO equipment readiness | 0.0841 |
|  | Rate of implementation of blood transport assessment of limb | 0.0459 |
|  | Rate of qualified ECMO pipeline maintenance | 0.0414 |
|  | Rate of implementation of assessment of neurological changes | 0.0267 |
|  | Rate of implementation of complication risk factor assessment | 0.0196 |
|  | Rate of implementing ECMO verification checklists | 0.0405 |
|  | Rate of implementation of transport safety of ECMO patients | 0.0286 |
|  | Rate of qualified pipe prefilling | 0.0245 |
|  | Rate of anti-coagulation target attainment | 0.0185 |
|  | Rate of analgesia and sedation reaching the standard | 0.0154 |
|  | Rate of execution of withdrawal test | 0.0097 |
|  | Rate of target temperature attainment | 0.0384 |
|  | Rate of target blood flow compliance | 0.0192 |
|  | Incidence of centrifugal pump failure | 0.0347 |
|  | Incidence of decreased oxygenator function | 0.0693 |
|  | Incidence of bleeding | 0.0281 |
|  | Incidence of limb ischemia | 0.0176 |
|  | Incidence of hemolysis | 0.0172 |
|  | Incidence of thrombosis | 0.0151 |
|  | Incidence of ECMO catheter bloodstream infection | 0.0093 |
|  | Rate of ECMO effective removal | 0.0080 |
|  | Patients’ and family members’ average satisfaction with nursing care | 0.0085 |
|  | Incidence of unplanned extubation in patients on ECMO | 0.0173 |
|  | Incidence of pressure injury in patients on ECMO | 0.0347 |
